# BioC-compatible full-text passage detection for protein–protein interactions using extended dependency graph

**DOI:** 10.1093/database/baw072

**Published:** 2016-05-11

**Authors:** Yifan Peng, Cecilia Arighi, Cathy H. Wu, K. Vijay-Shanker

**Affiliations:** ^1^Computer & Information Sciences, University of Delaware and; ^2^Center for Bioinformatics & Computational Biology, University of Delaware, Newark, DE 19716, USA

## Abstract

There has been a large growth in the number of biomedical publications that report experimental results. Many of these results concern detection of protein–protein interactions (PPI). In BioCreative V, we participated in the BioC task and developed a PPI system to detect text passages with PPIs in the full-text articles. By adopting the BioC format, the output of the system can be seamlessly added to the biocuration pipeline with little effort required for the system integration. A distinctive feature of our PPI system is that it utilizes extended dependency graph, an intermediate level of representation that attempts to abstract away syntactic variations in text. As a result, we are able to use only a limited set of rules to extract PPI pairs in the sentences, and additional rules to detect additional passages for PPI pairs. For evaluation, we used the 95 articles that were provided for the BioC annotation task. We retrieved the unique PPIs from the BioGRID database for these articles and show that our system achieves a recall of 83.5%. In order to evaluate the detection of passages with PPIs, we further annotated Abstract and Results sections of 20 documents from the dataset and show that an *f*-value of 80.5% was obtained. To evaluate the generalizability of the system, we also conducted experiments on AIMed, a well-known PPI corpus. We achieved an *f*-value of 76.1% for sentence detection and an *f*-value of 64.7% for unique PPI detection.

**Database URL:** http://proteininformationresource.org/iprolink/corpora

## Introduction

The protein–protein interaction (PPI) extraction task involves detection of statements of physical interactions between proteins. Many efforts have contributed to different aspects of PPI extraction from the biomedical literature; from PPI document classification to PPI or PPI method detection (1–5). In particular, the BioCreative V BioC task (Track 1) proposes to build a framework that allows different text mining tools to be seamlessly integrated into a pipeline for literature curation of protein interactions (both genetic and physical interactions) to be evaluated by BioGRID database curators (6, 7). Our team participated in this task by contributing to detecting passages with PPIs over full-text articles. This task is to “find passages describing physical PPIs” (7). Physical interactions may appear in single or several sentences. Thus, in this task one “passage” consists of one or more consecutive sentences, but it does not have to be a paragraph in the document.

Full-text articles by nature use various syntactic constructions for mentioning similar information. These textual variations can be problematic for relation extraction (RE) systems to account for. A central theme of this study is the hypothesis that the varied forms of PPI mentions are essentially due to certain syntactic structural complexities. By capturing these regularities, we can build a system where the extraction patterns can be kept simple.

**Figure 1.**
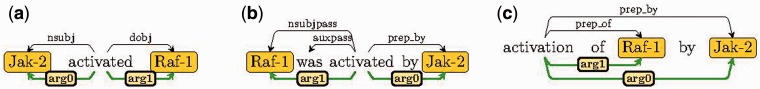
Sample EDGs with an (a) active, (b), passive (b), and (c) nominalized forms of the verb “activate”.

We have recently proposed a novel text representation, the extended dependency graph (EDG) that abstracts away certain text variations (8). EDG not only considers syntactic dependencies between words in a sentence, but also utilizes information beyond syntax to capture different dependencies. In particular, EDG adds numbered arguments in the dependency graph to provide consistent argument labels across different textual forms. For example, Figure 1 shows EDGs of three text fragments with syntactic edges above the words and numbered argument edges below. The numbered argument edges, *arg0* and *arg1*, unify the realization of active, passive and nominalized forms of the verb “activate” for purposes of PPI detection.

In the BioCreative V BioC task, the contribution of our project is to extend the framework for fast development of pattern-based biomedical RE, by incorporating the EDG. This intuition is partially based on our previous work that leverages syntactic variations in a language to achieve high precision (9), as well as the work that applies sentence simplification to improve the coverage of extracted relations (10–12). These two aspects, which are both incorporated in EDG, allow us to use only three sets of basic rules to detect PPI pairs. The BioC task is of PPI passage detection. Based on the detected PPI pairs, we pick the sentence that contains one or more PPI pairs. The additional rules are used to detect more passages over the full-text article by utilizing the detected PPI mentions.

We conducted three experiments to test the system. First, we retrieve all unique PPIs of 95 documents provided by the BioC track organizer from the BioGRID database. We evaluated our system on this dataset and achieve a recall of 83.5%. Second, since the task is passage detection and the annotations are not provided in the 95 document dataset, one of the authors (C.A.) annotated 20 full-text articles for this purpose. Experiments on these 20 in-house full-text articles show that we are able to obtain an *f*-value of 80.5% for PPI passage detection. Using these 20 documents, we also investigated a few heuristics to determine whether a detected PPI pair is experimentally validated. We obtained promising results with a precision of 81.4% and a recall of 91.5%. We plan to explore this issue further.

Third, to test the generalizability of our system as well as the precision of PPI detection, we evaluated the system on AIMed (13, 14), which is a widely-used PPI corpus. Since this corpus contains annotations with individual PPI mentions, we can use it for both PPI sentence detection and PPI pair detection. We obtained an *f*-value of 75.4% at a precision of 91.5% for the sentence detection and an *f*-value of 64.4% at a precision of 82.5% for the PPI pair detection.

**Figure 2.**
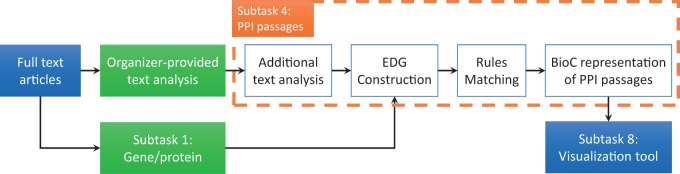
Flowchart of our system in the BioC subtask 4 pipeline.

Figure 2 summarizes that how our system (shown within the dashed lines) fits into the BioCreative V BioC task architecture (7, 15). Given the full-text articles, the organizers provided text analysis (such as sentence splitting), and the teams who participated in subtask 1 detected the gene/protein-named entities. Our system used these preprocessed full-text documents as input and produced BioC annotation indicating which sentence or block of sentences contains PPIs. This output was then used by teams who participated in subtask 8 for visualization. The collaborative framework used BioC format to transfer information, and was evaluated by the BioGRID curators to rate the usefulness of the whole integrated tool. The feedback from curators indicates that the performance of the curation tool is adequate to support the BioGRID curation task (7).

In our system, we parsed full-text articles to obtain the Stanford dependency graph of each sentence. Then, we constructed EDG from Stanford dependency graph and gene/protein-named entities. We used predicate-argument rules on EDG to extract PPI instances from sentences and additional rules to select passages with PPIs and finally produced PPI passages in BioC format. We will describe each of these steps in the following section.

## Methods

### Extended dependency graph

In general, all predicate-argument patterns rely on the edges, which link the trigger word and its two arguments (proteins). Due to a variety of reasons, articles use different forms of textual structures to express similar meanings. In many situations, it brings challenges to rule-based systems because a large number of rules are required to cover different text variations.

In this article, we use EDG to represent the structure of the sentence (8). The vertices in an EDG are labeled with information such as the text, part-of-speech and the word lemma. If an entity mention spans multiple tokens in a sentence, we merge their corresponding vertices into one vertex.

EDG has two types of dependencies. The syntactic dependencies are obtained from CCProcessed dependencies output by applying Stanford dependencies converter (16) on a parse tree obtained by Bllip parser (17). The other type of dependencies is the numbered arguments, whose idea is based on the guidelines of PropBank (14). For the PPI detection task, we use only *arg0* and *arg1* in EDG. To create *arg0* and *arg1* in EDG, we use different syntactic dependencies obtained from the Stanford typed dependencies. For verbal predicate, we mostly rely on syntactic dependencies such as “nsubj”, “nsubjpass”. For nominal forms, we investigated structures of noun phrases such as “interaction between *X* and *Y*” and “binding of *X* and *Y*”. In these cases, *arg0* and *arg1* are added between the nominalization of verbal predicates and its arguments based on syntactic dependencies like “prep_of” and “conj_and”. For the demonstration purpose, we show one construction rule below, which is used to create *arg0* and *arg1* edges in Figure 1b.

$$\begin{array}{lll} VBN\left(\text{verb}\right) & & \\ nsubjpass\left(\text{verb},\text{ entity1}\right) & \Rightarrow & arg1\left(\text{verb},\text{ entity1}\right)\\ prep\_by\left(\text{verb},\text{ entity2}\right) & & arg0\left(\text{verb},\text{ entity2}\right) \end{array}$$

Oftentimes, *arg0* and *arg1* link the nominal phrases that are not target protein mentions. For example, in “ARTS *binds* to a distinct domain in XIAP-BIR3”, *arg1* links “bind” to “domain”, but not “XIAP-BIR3”. For such cases, the EDG construction uses additional relations that are extracted. These relations are extra-syntactic and more semantic in nature. In the above example, the system first detects a part-whole relation from “domain” to “XIAP-BIR3, and then propagates *arg1* from “domain” to “XIAP-BIR3”. In general, if there is an *arg0* (*arg1*) edge from a node *n1* to *n2* and a part-whole edge from *n2* to *n3* then the propagation phase now adds an additional arg0 (*arg1*, respectively) edge from *n1* to *n3*. Thus we are able to get “ARTS ← *arg0* ← binds → *arg1* → XIAP-BIR3”. In addition to the *part-whole* relation, we also detect *is-a* indicating the relation between *X* and *Y* when *X* is a subtype of *Y*; *member-collection* indicating the link between a generic reference to a group of entities that specified in other places in the sentence; and co-reference, including abbreviation, indicating the relation between multiple expressions and one referent. More details on how *arg0* and *arg1* are created, how the four above-mentioned extra-syntactic relations are determined and propagated can be found in (8, 9).

Table 1 shows basic examples of sentence constructs that encapsulate a PPI but are expressed in different ways. Note that some examples in Table 1 are the combination of different constructs, but it is straightforward to identify the main construct in the examples.

**Table 1.** Constructs with examples

Type		Explanation	Example
1	Active form	Verbs in an active voice	**HFE** *binds* to the **transferrin receptor**
2	Passive form	Verbs in a passive voice	**Plasminogen activator inhibitor 1 (PAI)** is *bound* to **vitronectin** in plasma.
3	Nominalization	Nominalized verbs	*Binding* of **G beta gamma** to **Raf/330**
4	Adjective	Verbs used as an adjective	**Raf-1**-*binding* proteins, **Ras**
5	Full relative clause	Relative clauses introduced by relative pronouns, such as “which”, “who”, and “that”.	**Shc**, which specifically *binds* the SH2 domain of **GRB2**
6	Reduced relative clause	Relative clauses that start with a gerund or past participle and have no overt subject.	Structure of **ERK2** *bound* to **PEA-15** reveals a mechanism for rapid release of activated MAPK.
7	Coordination	Structures that link two or more items (conjuncts) of syntactically equal status.	**p53** [*binds* and activates]_coordination_ the **xeroderma pigmentosum DDB2 gene** in humans
8	Null argument	When the argument is omitted, but implied	**Histone deacetylase 1** can repress transcription by *binding* to **Sp1**.
9	Is-A	Argument X is a hyponym of argument Y, if X is a subtype of Y, or when an instance of X refers to a concept Y	**CD5** is a T-cell-specific antigen which *binds* to the B-cell antigen **CD72**
10	Appositive	Constructs of two noun phrases next to each other, typically separated by comma and referring to the same entity	**TPO** *binds* and activates its receptor, **myeloproliferative leukemia virus receptor**
11	Member-collection	Constructs that link a generic reference to a group of entities that are specified in other places in text.	The basic cleft of **RPA70N** *binds* multiple checkpoint proteins, including **RAD9**
12	Part-whole	Constructs that an argument extracted for a trigger comprises a part of the target entity.	**ARTS** *binds* to a distinct domain in **XIAP-BIR3**
13	Combination	Any combination of above types	**TR6** specifically *binds* two cellular ligands, **LIGHT (herpes virus entry mediator (HVEM)-L)** and **Fas ligand (FasL/CD95L)**

Entities in the extracted relations are marked in bold font.

**Figure 3.**
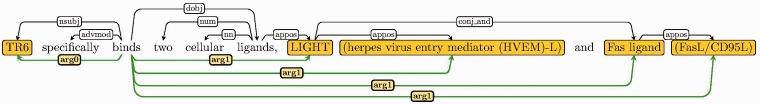
Sample EDGs with coordination and apposition.

For all sentences in Table 1, EDG, through its use of numbered argument labels and detection of different sentence structures, alleviates the textual variations challenge by mapping them to the same single base form. This base form, shown in Figure 3 as the EDG of the last example in Table 1, corresponds to each triple of arg0, arg1 edges and the trigger word they emanate from. Note that all constructs (one coordination and three appositions) contribute individual *arg1* representations. Each *arg0* and *arg1* pair then corresponds to a pair of interacting protein mentions.

### Basic predicate–argument rules with triggers

We use predicate–argument rules on EDG to extract PPI pairs. The predicate can be “bind”, “interact” or “crosslink” that triggers the potential occurrence of a PPI pair. The set of triggers were determined in consultation with the domain expert (CA) and also by examining the BioGRID guidelines (http://wiki.thebiogrid.org/doku.php/curation_guide). Additional triggers for binding were included after an investigation of BioNLP 2011 binding corpus. Since EDG applies lemmatization to abstract from different inflectional forms of words, only the common base forms of triggers (lemma) are used in the system. The complete list of all triggers can be found in the supplementary table (S1).

Because numbered arguments and their propagation provide a uniform representation for various textual variations (e.g. consider the range of sentences in Table 1), EDG allows the number of rules to extract PPIs to greatly reduce. In our system, only two sets of rules are used.

1. Direct trigger
  - Protein ← *arg0* ← PPI/PTM verb trigger → *arg1* → Protein
  - Protein ← *arg0* ← PPI/PTM noun trigger → *arg0* → Protein
2. Indirect trigger
  - Protein ← *arg1* ← process trigger ← *arg0* ← indirect trigger → *arg1* → Protein
  - Protein ← *arg1* ← indirect trigger → *arg1* → process trigger → *arg1* → Protein

Rule 1a is a set of most basic and strict rules. We use PPI triggers (e.g. “associate” and “bind”) and post-transcriptional modification triggers (e.g. “acetylate” and “methylate”) in the system. Because EDG has unified different forms of predicates in the vertices, we only need to list stems of triggers in the rules. Rule 1a employs trigger stems that are verbal but, of course, can match noun forms such as “association” in the text.

Rule 1b accounts for triggers that are not derived from verbs (e.g. “complex” and “dimer”). This rule matches the noun phrase such as “[*X*]_protein_–[*Y*]_protein_ complex”.

Rules 2a and 2b account for indirect PPI triggers such as “block” and “mediate”. These triggers connect a protein with an activity of another protein. In our system, the process triggers include “activity” and nominalization of PPI triggers whose suffixes are “-ion”.

Once an EDG is produced for a sentence, the above rules are matched with the EDG using a subgraph-matching algorithm (18). For each rule, a subgraph is constructed. Both nodes and edges in the subgraph are predicates of EDG nodes and edges. The worst-case complexity of the subgraph matching algorithm is *O*(*n*^2^*k^n^*) where *n* is the number of vertices in EDG and *k* is the vertex degree. It is worth noting that we only use *arg0* and *arg1* in the rules, thus EDG only contains numbered arguments, and the matching is efficient in practice.

### Non-predicate–argument rules to increase recall of passage detection

Our system uses EDG with basic rules to detect PPI interacting partners, and then selects the corresponding sentences. For the BioCreative V BioC task, we felt that other sentences that contain the detected protein pairs might also be the interest of biocurators. For example, if we are able to detect that “STRAP” and “Smad7” interact somewhere in the article with PMID 10757800, then we would also like to pick the sentences such as “We used both Flag- and HA-tagged STRAP and Smad7 in the coimmunoprecipitation experiments, demonstrating that the association was independent of the epitope tag employed and that the amino- or carboxy-terminal tags did not alter the association of the proteins.” in the same document. The basic rules in the previous section are not sufficient to extract any PPI information. However, under the assumption that such sentences will be useful for curation of PPI information, we have added two additional rules listed below. It is to be noted that these two rules are applied only to sentences containing two proteins, which are already known to interact somewhere else in the article. Thus, these rules only boost the recall of passages detection only and not that of the detection of interacting partners.

#### Experimental techniques with 2 proteins

To identify new PPI, experiments are conducted and described in the paper. Such description will be captured by our system when both the experimental technique and two (interacting) proteins are mentioned in the same sentence. Currently, we use only five technique keywords in our system: “2-hybrid”, “BIFC”, “cosedimentiation”, “ITC” and “pulldown”.

#### Extension with PPI triggers and 2 proteins

In some complicated sentences, the PPI triggers and two proteins are mentioned but there is no direct EDG edge between proteins and trigger nodes in EDG. This is especially true when this sentence is followed by a sentence where the interaction between these two proteins has been detected already. The hypothesis is that the block of sentences is a continuation of the same topic. The following text fragment (PMCID: PMC137860) shows such an example. In the first sentence (in the “Results” section), we extract the PPI pair <Kap β2B, *TAP*>. In the second sentence (in the “Discussion” section), no pattern can be applied. However, since both “interaction” and the pair appear, we extend the PPI passage to include the second sentence as well.

***Kap β2B*** *Is Associated with* ***TAP*** *in the Presence of RanGTP (1st sentence in the Results section)*

…

*The data presented support the conclusion that* ***Kap β2B*** *is a major carrier for export of cellular mRNA and* ***TAP*** *connects* ***Kap β2B*** *to the mRNAs to be exported, whereas the direct interaction of* ***TAP*** *with nucleoporins allows lower rates of mRNA export. (1st sentence in the Discussion section)*

## Evaluation and analysis

The participants in the BioC task were provided 120 articles by the organizers. Our first evaluation was based on the PPI information associated with these articles in the BioGRID database. Note, BioGRID only marks the unique PPIs discovered in the study described in the article. Hence, the articles could have included mentions of other PPI partners. Since our system is designed to extract all PPI pairs in text regardless of whether the pairs are validated in the experiments conducted by the authors, we cannot use this dataset to calculate the precision of the system but only calculate the recall (Table 2). Over the total 120 full-text articles, we observed that some have a large number of PPIs in a network in supplementary data, which were not found in the articles’ text shown (e.g. PMID 24711643). Such cases tended to be ones where a significant number of PPIs were associated with articles in BioGRID. For this reason, we only chose papers with 10 or fewer pairs for the evaluation (95 in total). Over these 95 full-text articles, our system was able to extract 263 unique pairs out of 315 marked in BioGRID, which yields 83.5% in recall.

**Table 2.** Recall on 95 annotated documents

	**TP**	**FN**	**Recall (%)**
Unique PPI	263	52	83.5

Note that while the BioC task was the detection of PPI passages rather than the PPI protein pairs, the data derived from BioGRID cannot be used for PPI passage detection evaluation. This is because BioGRID does not contain information about the passages from which PPI pairs are mentioned. Therefore, to further evaluate the system performance on passage detection, we created a test set ourselves. We randomly chose 20 articles from the 95 and one of the authors (C.A.), who is an experienced biocurator, annotated the Abstract and Results sections of this set of articles. Table 3 shows the results of PPI passage detection. On this 20 in-house datasets, we obtained 80.5% in *f*-value. We linked the dataset from http://proteininformationresource.org/iprolink/corpora.

**Table 3.** Recall on 20 in-house annotated documents (only Abstract and Results sections)

**Section**	**TP**	**FP**	**FN**	**Precision (%)**	**Recall (%)**	***F*-value (%)**
Abstract	20	5	4	80.0	83.3	81.6
Results	216	79	26	73.2	89.3	80.4
*Total*	*236*	*84*	*30*	*73.8*	*88.7*	*80.5*

Our system did not distinguish PPI passages that correspond to results experimentally validated in the article (“new” PPIs) versus passages that do not. However, curators are probably more interested in the former ones. While we have not fully developed the system to only identify passages with “new” PPIs, we consider a hypothesis about the position and structure of such passages. We believe that the passages that meet the following criteria will more likely discuss the experimental results of the article.

1. The Results section (if applicable) or the whole Abstract;
2. The titles of subsections in the Results section;
3. Figure captions in the Results section;
4. Any sentence that indicates the goals of experiments, such as beginning with “to investigate”.

To probe the effectiveness of these criteria, we limited our PPI pairs to true positives and then considered how many of those also appear in the BioGRID database. It is noteworthy that any false positives in PPI pair detection will obviously not appear in the BioGRID database, hence are not relevant for new PPIs detected in the document. Since we could only identify the true positive PPI pairs for the 20 in-house documents, we used these documents for our study. Of 65 true positive PPI pairs, 54 were annotated in the BioGRID database, giving a precision of 83.1%. There is a total of 59 distinct PPIs in the BioGRID database, five do not meet the criteria, giving a recall of 91.5%. In the future, we plan to investigate this issue further and extend our system to identify PPIs that are experimentally validated in the article.

We also apply the RE system on the AIMed corpus (13), which is commonly used in PPI extraction tasks and has been suggested by the task organizers as a training set (for machine learning systems). Table 4 reports two sets of performance metrics based on how we compare the system annotation with the gold standard. Since we applied the system at the sentence level, only basic rules were used to obtain both results (“Basic predicate–argument rules with trigger” section). The first row shows the performance of selecting sentences with PPI rather than the pairs. We conducted this experiment because our current task is for PPI passage detection rather than the PPI interaction detection. Similarly, we modified the AIMed annotations to indicate whether a sentence mentions a PPI or not. For the sentence selection task, we achieved an *f*-value of 76.1% at a high precision of 92.7%. The second row shows the performance of detecting PPI pairs, which is the traditional PPI extraction task. We obtained an *f*-value of 64.7%. It is noteworthy that we achieve these results by using just basic rules, and the results obtained are among the best obtained on this corpus. This shows the advantages brought out by the use of EDG and furthermore suggests the generalizability of the system since it was not developed specifically for the characteristics of AIMed.

**Table 4.** Evaluation results on AIMed

**Task**	**TP**	**FP**	**FN**	**Prec. (%)**	**Recall (%)**	***F*-value (%)**
Sentence detection	370	29	197	92.7	64.6	76.1
PPI pairs	557	165	443	77.2	55.7	64.7
Rule 1a	458	116				
Rule 1b	86	46				
Rule 2	13	3				

To analyze the contribution of each rule, we count how many TP and FP instances are extracted by basic rules. Rows 4–6 in Table 4 show that patterns with verb and noun triggers are able to extract 96% instances. On the other hand, indirect rules have extracted very few cases. This is partially because we did not consider general verbs as the indirect triggers due to high precision concerns. In future, we will include more trigger words to improve the recall. At the same time, to maintain the precision, we will propose restrictions to exam whether the trigger word indicates a “direct” interaction.

## Conclusion

In BioCreative V BioC task, we developed a PPI system to detect text passages with PPIs in the full-text articles. By adopting the BioC format, the output of the system could be added to the biocuration tool. The feedback from curators indicates that the performance of the curation tool is adequate to support the BioGRID curation task.

In addition, we evaluated the BioC-compatible PPI system on 95 documents for unique PPI detection, 20 in-house documents for passage detection and the widely used AIMed corpus for both unique PPI and sentence detection. All experiments confirm that the system is able to achieve good performance.

The development of our rule-based system is based on the semantic dependencies between entities that are critical for either pattern-based or machine learning systems. In this article, we show the use of a few rules on EDG still enables us to get good coverage of passage/PPI detection. This, in particular, allows us to address one of the main criticisms against rule-based systems—it is hard to develop rules for all the variations found in the text. We believe this information is not task dependent and an enhanced understanding will contribute to developing systems for various RE tasks, including genetic interactions defined in BioGRID in this track.

## Supplementary data

Supplementary data are available at *Database* Online. 

Supplementary Data
