# Supplementary material for: BioC-compatible full-text passage detection for protein–protein interactions using extended dependency graph
Source: Database (Oxford). 2016 May 11;2016:baw072. doi: 10.1093/database/baw072 (PMC4915133; doi:10.1093/database/baw072)
Supplement: Supplementary Data [file supp_2016_baw072_index.html]

Supplementary Data 

# BioC-compatible full-text passage detection for protein–protein interactions using extended dependency graph

## Supplementary Data

files

- Supplementary Data - doc file
