# Supplementary material for: BioC-compatible full-text passage detection for protein–protein interactions using extended dependency graph
Source: Database (Oxford). 2016 May 11;2016:baw072. doi: 10.1093/database/baw072 (PMC4915133; doi:10.1093/database/baw072)
Supplement: Supplementary Data [file supp_baw072_Supplementary_material.doc]

# Supplementary material

**S1. Trigger list.**

| **Trigger** | **Stem** |
| --- | --- |
| Verbal PPI trigger | activate (co-, in-) |
|  | associate (co-, dis-, re-) |
|  | bind |
|  | cleave |
|  | crosslink |
|  | immunoprecipitate (co-) |
|  | interact |
|  | recruit |
|  | acetylate (de-) |
|  | glycosylate |
|  | methylate |
|  | phosphorylate (auto-, de-) |
|  | ubiquitinate |
| Noun-based complex trigger | complex |
|  | dimer (heter-, homo-) |
|  | heteromer |
|  | homomer |
|  | homotrimer |
|  | oligomer |
|  | trimer |
| Process trigger | activity |
|  | v-ion |
| Indirect regulatory trigger | block |
|  | decrease |
|  | impair |
|  | induce |
|  | mediate |
|  | regulate |
|  | repress |
|  | require |
|  | restrict |
|  | stimulate |
|  | trigger |

**S2. Technique word/phrase list**

2 hybrid

2-hybrid

bifc

cosedimentation

ITC

itc

pull down

pull-down

pulldown

two hybrid

two-hybrid
